# Fine Structure and the Huge Zero-Field Splitting in Ni^2+^ Complexes

**DOI:** 10.3390/molecules27248887

**Published:** 2022-12-14

**Authors:** Miroslav Georgiev, Hassan Chamati

**Affiliations:** Institute of Solid State Physics, Bulgarian Academy of Sciences, Tzarigradsko Chaussée 72, 1784 Sofia, Bulgaria

**Keywords:** single-ion magnet, zero-field splitting, exact diagonalization, spin–orbit coupling, spin exchange interactions

## Abstract

We perform a thorough study of the ground state magnetic properties of nickel-based $3d^{8}$ complexes. This includes an in-depth analysis of the contribution of the crystal field, spin exchange and spin–orbit interactions to the ground state magnetic properties. Of particular interest to the current investigation are the presence and occurrence of non-trivial zero-field splitting. The study focuses on the cases of Ni${}^{2+}$ ideal octahedral, trigonal bipyramidal, square planar and tetrahedral geometries. We provide results for the complete energy spectrum, the fine structure related to the ground state and the second set of excited states, low-field magnetic susceptibility and magnetization. In addition, we examine the zero-field fine structure in square pyramidal, trigonal pyramidal and trigonal planar complexes. The obtained results unequivocally show that a moderate or highly coordinated $3d^{8}$ complex can neither exhibit spin–orbit-driven large and giant magnetic anisotropy nor a huge zero-field splitting. Moreover, in the trigonal bipyramidal coordination, a fine structure associated to the ground state cannot result from the spin-orbit coupling alone.

## 1. Introduction

Since the establishment of molecular nanomagnetism as a standalone research field, the quest of advanced technology to engineer the magnetic properties on a micro- and nanoscale has driven the experimental and theoretical investigations on molecular nanomagnets to unprecedented levels [1,2,3,4,5,6,7,8,9,10,11]. On the smallest scale possible for manipulation, the deposition of single or polyatomic units on a surface [12,13,14,15,16,17] and the synthesis of isolated polynuclear [18,19,20,21,22,23,24,25] and mononuclear [26,27,28,29,30,31] magnetic units that may display a strong anisotropy enjoy an ever-growing interest. Assembling single-molecule and single-ion magnets that possess a considerably large temperature-resistant energy barrier to magnetization reversal (switching) is a great challenge in front of their potential application as magnetic information storage devices. Such a progress is inextricably related to extracting useful knowledge about the origin of the underlying fine structure (FS) and corresponding magnetic anisotropy (MA).

Over the last two decades, the fine structure of the ground state (FSG) of mononuclear $3d^{8}$ systems has been a subject of great interest [32,33,34,35,36,37,38,39,40,41,42,43,44]. Among the complexes exhibiting small or moderate zero-field splitting (ZFS) [32,33,42,44,45,46,47,48], those showing a signature of non-trivial ZFS are the most challenging for experimentalists and theorists alike.

Recently, experimental observations [35] for huge energy gaps within FS in the trigonal bipyramidal complexes [Ni(Me${}_{6}$tren)Cl]ClO${}_{4}$ and [Ni(Me${}_{6}$tren)Br]Br have become available. All findings are assumed to be governed by a non-vanishing, even in the case of low symmetry, first-order spin–orbit coupling that emanates from the high degeneracy of the ground state related to the $d_{xy}$ and $d_{x^{2}-y^{2}}$ orbitals. In other words, the huge ZFS is expected to take place due to a non-negligible unquenched orbital angular momentum. Under the same theoretical assumptions, Craig et al. [41] studied the zero-field FSG in the trigonal bipyramidal complex [Ni(MeDABCO)${}_{2}$Cl${}_{3}$]${}^{+}$, obtaining very large values for the axial ZFS parameter. In an attempt to gain deeper insight into the possible occurrence of huge ZFS in Ni${}^{2+}$ complexes, Gómez-Coca et al. [36] performed an investigation in FSG under the same assumption—the unquenching of orbital momentum. They studied the trigonal pyramidal compound K{Ni(N[CH${}_{2}$C(O)NC(CH${}_{3}$)${}_{3}$]${}_{3}$)}, obtaining a very large in magnitude axial ZFS parameter (see also Ref. [49]). Similarly, the reported values for the axial ZFS parameter calculated for different Ni${}^{2+}$-based complexes [37] are directly addressed by the intrinsic value of the spin–orbit coupling. Furthermore, the experimentally observed field-induced barrier and ZFS properties of the trigonal bipyramidal complex [Ni(MDABCO)${}_{2}$Cl${}_{3}$]ClO${}_{4}$ are studied under the same assumptions [50].

The nascent unquenched orbital angular momentum and hence a first-order spin-orbit contribution, however, are not allowed by the Pauli exclusion principle in highly coordinated $3d^{8}$ complexes. That makes the unquenching case arguable and the correct evaluation of the spin–orbit contribution under the strong crystal field (CF) in such systems substantial for untangling the cases of huge ZFS. Therefore, the absence of a comprehensive theoretical investigation of FSG in $3d^{8}$ complexes raises basic questions regarding the genuine origin of all magnetic excitations in trigonal bipyramidal Ni${}^{2+}$ complexes, the determination of ZFS and single-ion anisotropy (SIA).

The very same questions follow from the outcome of huge ZFS in Ref. [38]. A more recent analysis [39] of ZFS to the ground state of the compounds [Ni(Me${}_{6}$tren)Cl]ClO${}_{4}$ and [Ni(Me${}_{6}$tren)Br]Br discussed the possibility of observing very large ZFS and the need of more rigorous and extensive investigation on the contribution and interplay of CF and spin–orbit interaction terms. The study, furthermore, discusses the misinterpretation of ZFS parameters as magnetic anisotropy ones, adding a very important clarification on the difference and relation between ZFS and SIA. For further information about the crucial notions, such as ZFS and SIA, the reader may consult Refs. [1,2,51,52,53,54,55,56].

Despite all past efforts to elucidate the occurrence of ZFS in some Ni${}^{2+}$ trigonal bipyramidal complexes, the possible emergence of huge ZFS are not fully clarified yet. To this end, computational techniques that provide a thorough understanding of the correspondence between the initial antisymmetric electronic quantum states and the obtained FS are required. To the best of our knowledge, a study based entirely on an exact diagonalization method has not yet been used to investigate the ZFS in $3d^{8}$ systems.

In the present study, we provide a thorough investigation of the contribution of CF, spin exchange and spin–orbit interactions on FS of nickel-based $3d^{8}$ complexes possessing different geometric symmetries with the aid of the theoretical framework proposed in Ref. [57]. The named approach is based on the exact diagonalization of the full Hamiltonian of the complex under consideration, making use of the fact that the core electrons do not affect the corresponding energy level sequence and hence do not alter the complex’s magnetic behavior. We emphasize on the ground state magnetic properties of Ni${}^{2+}$ ideal octahedral, trigonal bipyramidal, square planar and tetrahedral complexes. In addition, we provide results for FSG in square pyramidal, trigonal pyramidal and trigonal planar complexes. For comparison, we calculate ZFS and magnetization-reversal barriers of V${}^{3+}$ complexes of the relevant coordination. Our results demonstrate that for moderate and highly coordinated $3d^{8}$ complexes, the spin–orbit coupling alone cannot generate huge ZFS and giant MA. Moreover, FSG in the case of trigonal bipyramidal coordination does not hold. Nevertheless, the calculations and relevant analysis suggest that the occurrence of huge ZFS is not impossible. For the latter to occur, intrinsic constraints over the phases of unpaired electrons’ orbital states must be imposed. The applied exact diagonalization approach has the advantage of taking into account such constraints and leads to a self-consistent multi-configurational method and comes with the advantage of knowing the explicit representations of all energy eigenstates in a given basis set of complete antisymmetric wave functions describing the electronic system. This allows one to gain knowledge on the correspondence between the obtained FS and the probability to observe each one of the initial quantum basis states and hence their interference. Consequently, one may directly distinguish the conventional ZFS pattern among other splitting ones to study the SIA with the corresponding relaxation processes and to trace back the origin of any related effect. Thus, in the present study, we examine closely the role of CF, spin exchange and spin–orbit interactions in the ground state magnetic properties to ensure the correct determination of ZFS and MA/SIA.

The rest of the paper is organized as follows. Section 2 briefly introduces the theoretical approach used in the study as well as all relevant physical quantities. A representation of the Hamiltonian and all relevant interaction terms is provided. Section 3 reports results on ZFS and magnetic properties of the most common Ni${}^{2+}$ coordination geometries. Our main results are outlined in Section 4.

## 2. Theoretical Background

### 2.1. General Considerations

To study the magnetic properties of $3d^{8}$ complexes and in particular the ground state ones, we employ the exact diagonalization method introduced in Ref. [57]. Within the named theoretical approach, the $3d^{8}$ complexes are viewed as effective spin-one systems comprising eight 3d electrons and a number of point-like charges associated to each metal ion, with the directly bonded reactive non-metals being constituents of the relevant ligands. Since the eight electrons are considered localized in the vicinity of the metal ion, the active space is restricted to the number of 3d orbitals. In general, the orbital and spin magnetic dipole–dipole interactions associated to all electrons and the constituent nuclei have a negligible contribution to the FS under investigation. As a result, they are not accounted for in the calculations. Similarly, the hyperfine interactions are also omitted.

Here we introduce the mathematical definitions of all physical quantities used throughout the rest of the manuscript. The *i*-th electron spin and orbital angular momentum operators are denoted by ${\hat{s}}_{i}={\left({\hat{s}}_{i}^{\alpha }\right)}_{\alpha \in K}$ and ${\hat{l}}_{i}={\left({\hat{l}}_{i}^{\alpha }\right)}_{\alpha \in K}$, respectively, where K={x,y,z}. The expectation value of the total magnetic moment operator associated to the *n*-th energy level is denoted by $\mu_{n}={\left(\mu_{n}^{\alpha }\right)}_{\alpha \in K}$, with $\mu_{n,s}$ and $\mu_{n,l}$ representing the corresponding spin and orbital angular momentum components. The magnetization per single complex is denoted by $M={\left(M_{\alpha }\right)}_{\alpha \in K}$, with |M|=M. Note that the values of all magnetic moments given in the text are written in units of Bohr magneton $\mu_{B}$ and the electron’s *g*-factor is denoted by $g_{e}$. We have $d_{i}$ designating the *i*-th ligand position vector, with magnitude $d_{i}$, and $Z_{i}$ denoting the corresponding charge number. The charge number of the metal ion is *Z*. The externally applied magnetic field is denoted by $B={\left(B_{\alpha }\right)}_{\alpha \in K}$, with magnitude |B|=B. The elementary charge, electric and magnetic constants are denoted as *e*, ε and $\mu_{o}$, respectively. We select the *z* axis as the quantization axis and use “$\bar{1}$” instead of “−1” in the bra-ket notation of the spin states. The energy levels are denoted by $E_{i}$, with i=1 corresponding to the ground state one, and are normalized such that $E_{1}$ equals zero. The quantities $U_{\mathrm{eff}}$ and $U_{\mathrm{eff}}^{f}$ denote, respectively, the zero-field and field-induced magnetization-reversal barriers of an isolated complex. In particular, $U_{\mathrm{eff}}^{f}$ is the energy barrier between the fully polarized magnetic states of a single complex taken with respect to its quantization axis. In the calculations of the susceptibility, the ratio between the molar mass and density is taken as unity.

Let us note that the calculations for all studied coordination complexes are performed for the same values of the bond distances and charge numbers. Thus, we have $d_{i}=2$ Å, $Z_{i}=1$, Z=12 and the covalence factor κ=1, where, depending on the geometry, 3≤i≤6. Here, the value of *Z* is chosen to represent the Ni${}^{2+}$. Finally, the probability coefficients to all eigenstates are taken with an accuracy of $10^{-2}$.

### 2.2. The Hamiltonian

The Hamiltonian describing a $3d^{8}$ coordination complex within the considered active space reads

(1) $$\hat{H}={\hat{U}}_{R}\left(r_{1},\;\ldots \;,r_{8}\right)+{\hat{U}}_{\mathrm{CF}}\left(r_{1},\;\ldots \;,r_{8}\right)+{\hat{U}}_{\mathrm{SO}}\left(r_{1},\;\ldots \;,r_{8}\right)+{\hat{U}}_{Z},$$

where $r_{i}={\left(\alpha_{i}\right)}_{\alpha \in K}$ is *i*-th electron position vector, the operator accounting for the Coulomb interactions is given by

(2a) $${\hat{U}}_{R}\left(r_{1},\;\;\ldots \;,\;r_{8}\right)=\frac{1}{2}\sum_{1\leq i\neq j\leq 8}\frac{\gamma }{|r_{i}-r_{j}|},\;\gamma =\frac{e^{2}}{4\pi \varepsilon }.$$

The CF operator, accounting for the interactions of all 3d electrons with the effective ligands, is written as

(2b) $${\hat{U}}_{\mathrm{CF}}\left(r_{1},\;\;\ldots \;,\;r_{8}\right)=\sum_{k}\sum_{1\leq i\leq 8}\frac{\gamma Z_{k}}{|r_{i}-d_{k}|},$$

where *k* runs over the number of all ligands. The operator

(2c) $${\hat{U}}_{\mathrm{SO}}\left(r_{1},\;\;\ldots \;,\;r_{8}\right)=\frac{g_{e}\mu_{o}\mu_{B}^{2}}{2\pi }\sum_{1\leq i\leq 8}\frac{Z}{|r_{i}{|}^{3}}{\hat{l}}_{i}\cdot {\hat{s}}_{i}$$

represents the spin–orbit interactions of relativistic origin and

(2d) $${\hat{U}}_{Z}=-\mu_{B}\sum_{1\leq i\leq 8}B\cdot \left({\hat{l}}_{i}+g_{e}{\hat{s}}_{i}\right)$$

takes into account the interaction of all 3d electrons with the externally applied magnetic field. We would like to stress that all remaining interactions and kinetic terms entering into the full Hamiltonian of a complex are not included in (1) since the respective average is a constant that vanishes on account of the normalization of all energy eigenvalues.

Following Ref. [57], we work in spherical coordinates and expand the Coulomb (2a) and the crystal field (2b) components of Hamiltonian (1) in terms of Legendre polynomials. In deriving the relevant matrix elements and thus computing the magnetic properties of the compound under consideration, we truncated the ensuing expansions to the 4th order since this approach proved enough to capture the essential physics. Thus, the initial basis states, say $|\phi_{i,s,m}\rangle$ with total effective spin s=0,1 and magnetic number m=±s, representing the total antisymmetric wave function of all 3d electrons and used in the exact diagonalization, are combinations of the single electron CF states $|d_{xy}\rangle$, $|d_{xz}\rangle$, $|d_{yz}\rangle$, $|d_{z^{2}}\rangle$ and $|d_{x^{2}-y^{2}}\rangle$ in the bra-ket notation. More details are outlined in the Supplementary materials (Equations (S1) and (S2)).

We would like to emphasize that the calculation of the direct spin exchange interactions taking into account the effect of CF requires predefined local phases that relate the free-ion antisymmetric functions with the used ones (see Equation (S1)) by ensuring the same results regardless of the choice of the initial basis set. These phases are related to the dynamical electrons’ correlations and cannot be explicitly expressed in the used stationary initial basis states. However, following the method from Ref. [57] the corresponding quantitative effect is already accounted for (see Supplementary material, Equation (S3)). Thus, it is worth mentioning that the constraints over some phases may change completely the contribution of some interaction terms to FS of the resulting energy spectrum. A case showing how such a mechanism may effectively contribute to the magnetic properties of highly coordinated lanthanide single-ion magnets while occupied to a large extent *f* subshell is introduced in Ref. [58].

## 3. Coordination Geometries

### 3.1. Octahedral

**Figure 1:**
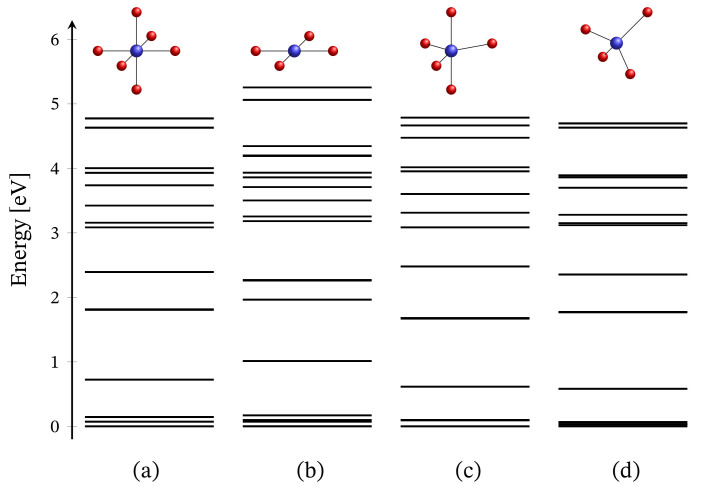
Zero-field energy spectra of four Ni${}^{2+}$ coordination complexes. Subfigures (**a**–**d**) represent the cases of octahedral, square planar, trigonal bipyramidal and tetrahedral geometry, respectively. The values of all parameters are given at the end of Section 2.1. At the given energy scale, each line represents a set of sublevels. The bolder a line, the larger the number of sublevels; see the numerical representation of these spectra in the Supplementary materials, Section S6.

**Figure 2:**
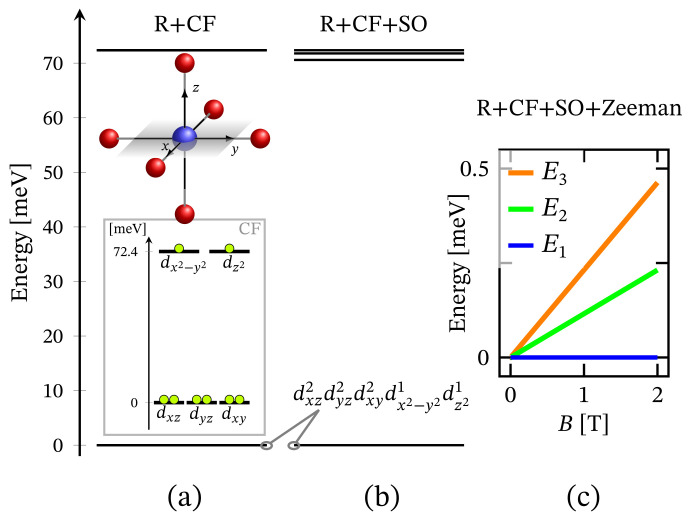
Low-lying energy level structure for an octahedral geometry, with model parameters given at the end of Section 2.1. (**a**) Shows the splitting occurring due to the action of CF under the repulsive Coulomb interactions. The corresponding CF orbital energy diagram is also depicted, where the lime colored circle represent an electron. (**b**) Depicts the added contribution of spin-orbit interactions. Subfigure (**c**) shows the Zeeman splitting.

The energy spectrum of the octahedral complex calculated by diagonalizing Hamiltonian (1) taking into account the relevant symmetry in the absence of external magnetic field is depicted in Figure 1a. The corresponding low-lying energy level sequence is shown in Figure 2b. As expected, there is no sign of ZFS. Thus, we have a 3-fold degenerate ground state, associated to the configuration $d_{xz}^{2}d_{yz}^{2}d_{xy}^{2}d_{x^{2}-y^{2}}^{1}d_{z^{2}}^{1}$. A fine structure, however, is observed only with respect to the first group of excited states. Actually, the first energy gap of approximately 70.6 meV, depicted in Figure 2b, results mainly under the action of CF, and this gap is about 72.3 meV or approximately 583.14 cm${}^{-1}$ when the spin orbit contribution is overlooked. The relevant energy levels are shown in Figure 2a. Any transitions related to the spectrum shown in Figure 2b are mainly governed by CF and should not be misinterpreted as a huge ZFS. It is worth mentioning that the calculated population of the first few excited states, assuming a powder sample, is about 1.8% at room temperature.

We would like to point out that the lack of FSG is observed for arbitrary type of ligands and even for a distorted geometry, except the cases of highly elongated and compressed bonds. Other exceptions are the complexes possessing pseudo-octahedral geometry [45] and distorted trigonal prismatic ones [33]. A case study showing how elusive the determination of ZFS could be is presented in Ref. [38]. Even for polynuclear $3d^{8}$ systems, such as the molecular magnet Ni${}_{4}$Mo${}_{12}$ [59,60,61], the evaluation of ZFS could be subtle. Our exact diagonalization results suggest that, due to the almost completely filled *d* subshell of the nickel ion, the contribution of spin–orbit interactions to the ground state properties of any ideal or distorted octahedral complex is zero.

**Figure 3:**
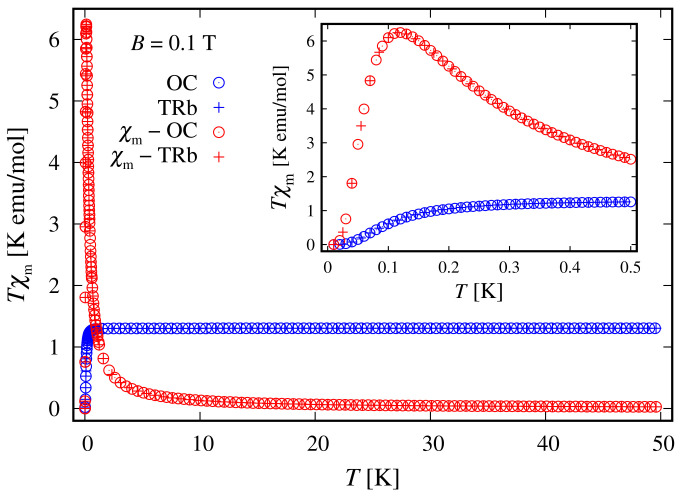
Simulated low-field molar susceptibility for the octahedral (OC) and trigonal bipyramidal (TRb) cases as a function of the temperature, with the parameter’s values specified at the end of Section 2.1. The ratio between the corresponding molar mass and density is taken as unity. The inset depicts the low-temperature domain.

**Figure 4:**
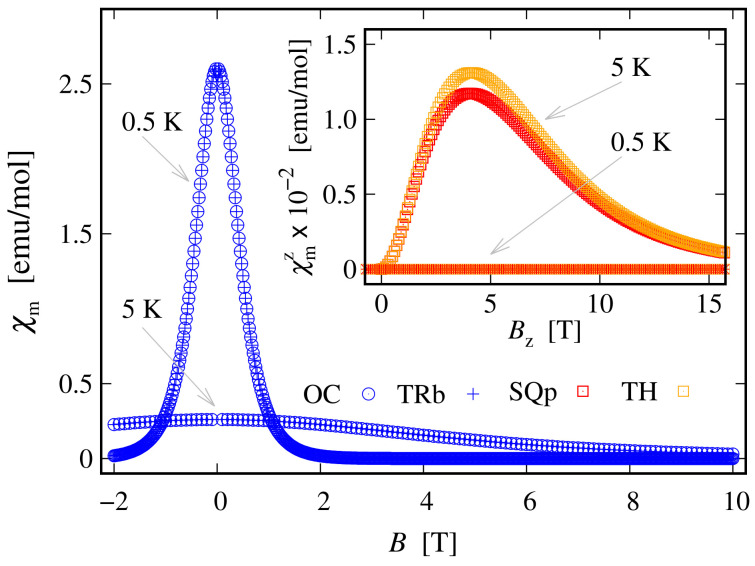
Low-temperature molar susceptibility as a function of the externally applied magnetic field for the octahedral (OC), trigonal bipyramidal (TRb), square planar (SQp) and tetrahedral (TH) complexes. The calculations are performed for parameters’ values given at the end of Section 2.1 and with a fraction between the corresponding molar mass and density equal to unity. The inset shows how the effect of raising the temperature to 5 K in the case of square planar and tetrahedral complexes affects the susceptibility. For convenience, only the *z* component of susceptibility is depicted. In particular, at T=5 K the peaks corresponding to $\chi_{m}^{x}$ and $\chi_{m}^{y}$ shift, with centers of approximately 3 T.

**Figure 5:**
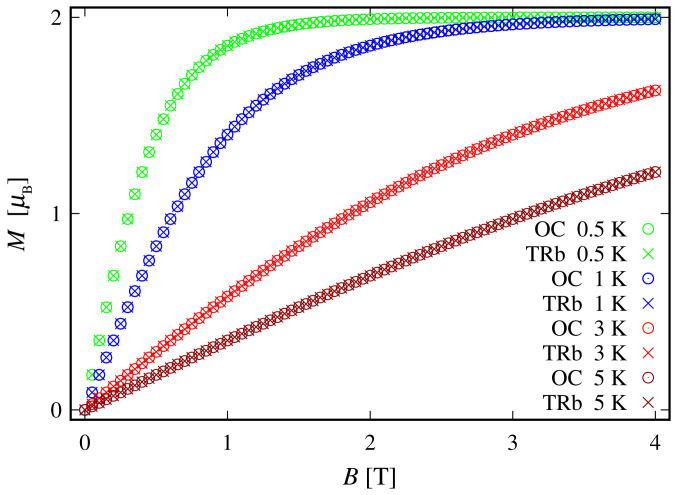
The magnetization of the octahedral (OC) and trigonal bipyramidal (TRb) complexes for different temperatures. The results are obtained for values of the model parameters provided at the end of Section 2.1.

Since the zero-field ground state energy is three-fold degenerate, the magnitude of the complex’s total magnetic moment is practically zero. In the presence of an external magnetic field, the compound is readily magnetized, thus exhibiting a genuine Zeeman splitting, see Figure 2c, with no orbital contribution. The paramagnetic behavior of this complex is shown in Figure 3, with low-field susceptibility reaching its maximum at approximately 0.1 K, for B=0.1 T. The same isotropic magnetic behavior shows up in the field dependence of the susceptibility depicted in Figure 4, with the center of its peak at the zero field’s value. For some low temperatures, the magnetization per single ion as a function of *B* is depicted in Figure 5.

### 3.2. Square Planar

**Figure 6:**
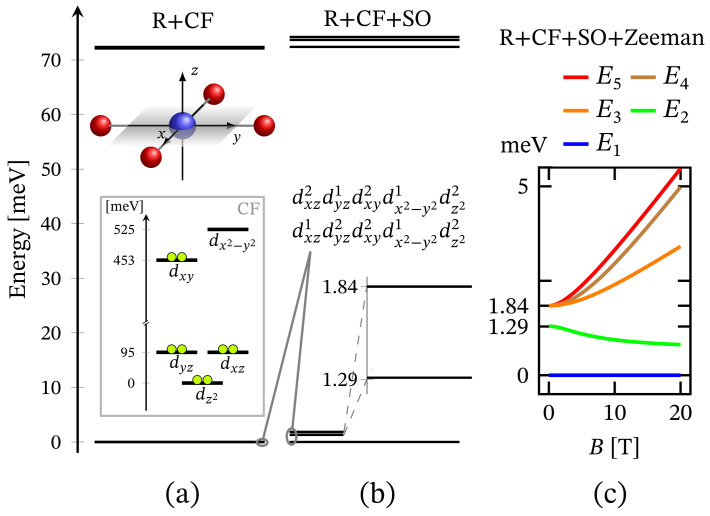
Low-lying energy level sequence in the case of square planar geometry, with parameters given in Section 2.1. (**a**) Depicts the splitting resulting from the action of CF along the repulsive Coulomb interactions. CF orbital splitting, with the respective electrons’ (circles) occupation, is shown in the inset. (**b**) Shows the added effect of spin-orbit contribution. (**c**) Presents FS in the presence of magnetic field applied along the *z* axis of the local reference frame.

The total energy spectrum in the case of square planar coordination obtained with the aid of Hamiltonian (1) obeying the associated structural symmetry at B=0 is shown in Figure 1b, with low-lying levels depicted in Figure 6b. In contrast to CF orbital energy diagram and associated configuration shown in the inset of Figure 6a, the ground and excited energy levels from the obtained FSG are associated with the combination of both $d_{xz}^{1}d_{yz}^{2}d_{xy}^{2}d_{x^{2}-y^{2}}^{1}d_{z^{2}}^{2}$ and $d_{xz}^{2}d_{yz}^{1}d_{xy}^{2}d_{x^{2}-y^{2}}^{1}d_{z^{2}}^{2}$ configurations. The difference comes as result of the Coulomb interactions (see Supplementary materials, Equation (S3)) that act in favor of the orbitals $d_{xz}$, $d_{yz}$ and $d_{x^{2}-y^{2}}$ as being active ones.

**Figure 7:**
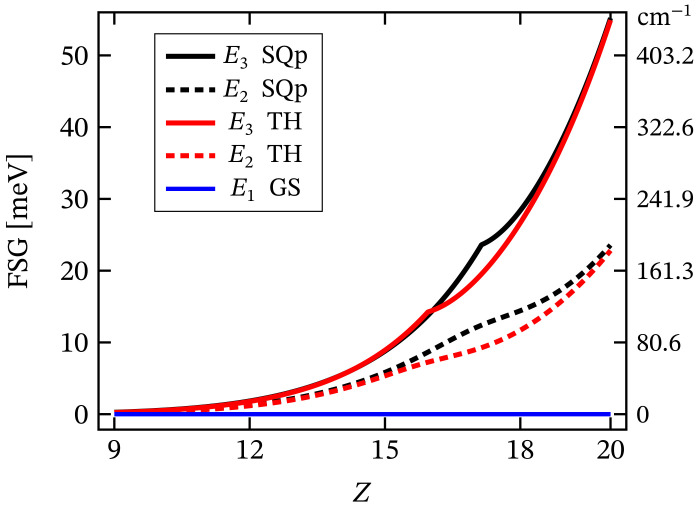
Zero-field FSG of the square planar (SQp) and tetrahedral (TH) geometries as a function of the metal ion’s charge number *Z*. Here, the abbreviation GS stands for ground state. The values of all relevant model parameters are given at the end of Section 2.1. More details about the values of first two excited energy levels in the case Z=12 are depicted in Figure 6b.

The value of the overall ZFS rapidly decreases while decreasing the metal ion’s charge number. As an example, for Z=10, its value is approximately four times smaller than that shown in Figure 6c. Hence, it drops from 1.84 to 0.53 meV. The non-linear dependence of ZFS on the metal’s charge number is displayed in Figure 7. On the other hand, the dependence of ZFS on κ is linear. Additionally, the calculations show that the square pyramidal geometry also exhibits FSG. However, neither the behavior of the square planar nor that of the square pyramidal complexes show signs of huge ZFS or giant MA. Actually, MA/SIA takes place only under the action of externally applied magnetic field, see Table 1, whereas in the absence of a magnetic field, the MA behavior is entirely suppressed by a tunneling between both spin-one fully polarized magnetic states. Concerning the tunneling effect, the reader may consult Ref. [62] and the reference therein.

At the zero magnetic field, the total magnetic moment obtained within FSG is a zero vector. In the case B≠0, this complex behaves like a single-ion diamagnet. This behavior is a consequence of the formation of two spin-one quadrupoles (see Supplementary materials, Equation (S6)) effectively represented by the states [63,64,65]

(3) $$|q_{x}\rangle =\frac{i}{\sqrt{2}}\left(|1,1\rangle -|1,\bar{1}\rangle \right),\;|q_{y}\rangle =\frac{1}{\sqrt{2}}\left(|1,1\rangle +|1,\bar{1}\rangle \right),$$

with ${\hat{s}}_{\alpha }|q_{\beta }\rangle =i\varepsilon_{\alpha \beta \gamma }|q_{\gamma }\rangle$, where $\hat{s}={\left({\hat{s}}_{\alpha }\right)}_{\alpha \in K}$ is the corresponding effective spin-one operator and $\varepsilon_{\alpha \beta \gamma }$ is the Levi–Civita symbol. The calculations show that the probability coefficients associated to these spin quadrupoles depend weakly on the variations of an externally applied magnetic field that enforces their stability.

**Figure 8:**
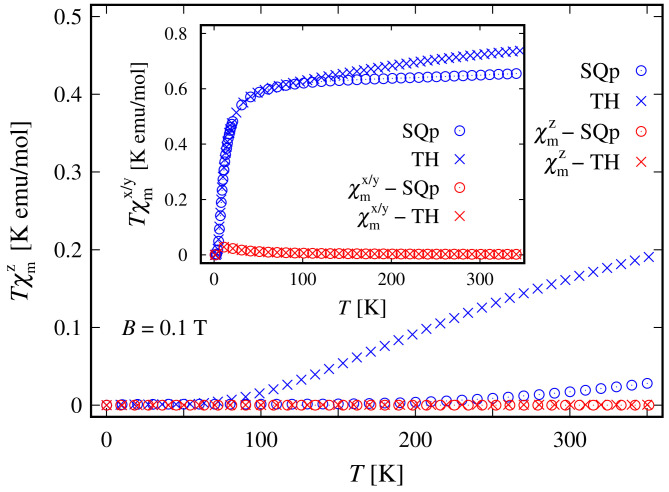
Low-field molar susceptibility as a function of the temperature in the case of a square planar (SQp) and tetrahedral (TH) geometries. The data are extracted for parameters given at the end of Section 2.1 and the ratio between the corresponding molar mass and density is taken as unity. In the inset, the superscript x/y stands for both the *x* and *y* components.

**Figure 9:**
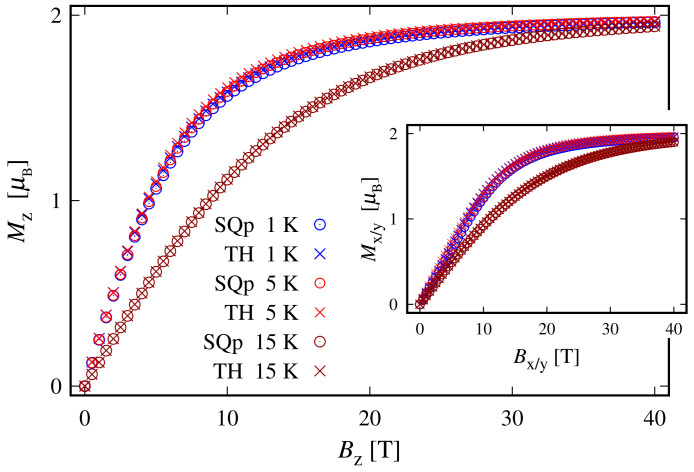
The magnetization of the square planar (SQp) and tetrahedral (TH) complexes for different temperatures. The values of all model parameters provided at the end of Section 2.1. The subscript x/y in the inset represents both *x* and *y* components.

The weak response to the externally applied magnetic field at low temperatures is evident from the dependence of the susceptibility on *T* and *B* depicted in Figure 8 and in the inset of Figure 4, respectively. At a low field along the *z* axis and within the whole temperature domain, the compound is not magnetically active. A weak activity is observed at 5<T≤30 K only when the field is parallel to the xy plane; see the inset in Figure 8. The susceptibility is identically zero even at higher fields; see the shallow peak depicted on the inset in Figure 4. The remarkable resistance to the action of external magnetic field, demonstrated by this complex, is further visible in the magnetization data depicted in Figure 9. The corresponding saturation of the magnetization is reached at $B_{\alpha }\geq 35$ T, where α∈K. Notice that in accordance to the susceptibility peak at 5 K, shown in Figure 4, the magnetization behavior at 5 K is slightly enhanced. A prominent feature of the magnetic property of this complex is that the saturation of the magnetization persists at temperatures as high as T=15 K.

### 3.3. Trigonal Bipyramidal

**Figure 10:**
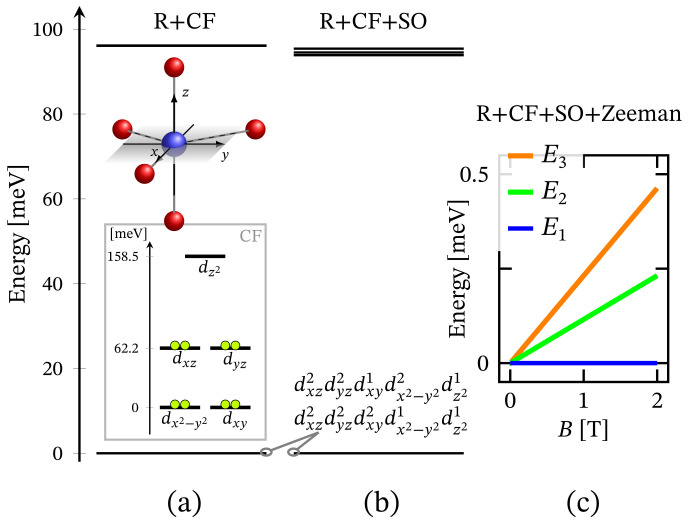
Low-lying energy level sequence for the trigonal bipyramidal coordination. The model parameters are given at the end of Section 2.1. (**a**) Shows CF splitting all repulsive Coulomb interactions accounted for. The orbital energy diagram and the corresponding electrons’ (circles) configuration resulting from CF is shown in the inset. (**b**) Depicts the spectrum with the contribution of spin–orbit interactions. (**c**) Shows the Zeeman splitting resulting from the action of the externally applied magnetic field.

The energy spectrum in the case of trigonal bipyramidal geometry computed by diagonalizing Hamiltonian (1) with the symmetry of this structure is illustrated in Figure 1c. A zero-field low-lying energy level sequence is depicted in Figure 10b, with a ground state represented by a superposition of the configurations $d_{xz}^{2}d_{yz}^{2}d_{xy}^{2}d_{x^{2}-y^{2}}^{1}d_{z^{2}}^{1}$ and $d_{xz}^{2}d_{yz}^{2}d_{xy}^{1}d_{x^{2}-y^{2}}^{2}d_{z^{2}}^{1}$. As in the case of octahedral coordination, here also a zero-field FSG is not observed. The obtained energy gap of 93.88 meV results mainly due to the action of CF. It is worth noting that in the absence of spin–orbit interactions, this gap is about 96.13 meV. This is shown in Figure 10a, where CF orbital splitting is also introduced. As a result of the selected short axial bonds, the energy of $d_{xz}$ and $d_{yz}$ orbitals is larger than that of the $d_{xy}$ and $d_{x^{2}-y^{2}}$ ones. In the case of slightly elongated axial bonds, it is the other way around. Nevertheless, since these types of diagrams are obtained without accounting for the matrix elements $\langle \phi_{i,s,m}|{\hat{U}}_{R}|\phi_{i,s,m}\rangle =E_{i,s}$, see Equation (S3) in Supplementary materials, the depicted configuration is not the ground state one. With the consideration of Coulomb repulsion between all eight electrons, the ground state configuration changes in favor of $d_{x^{2}-y^{2}},d_{z^{2}}$ and $d_{xy},d_{z^{2}}$ orbitals as active ones.

The effect of distortion on this type of geometry is prominent and may result in additional CF splitting between both ground state *d* configurations, but no ZFS. In general, the calculations suggest that even in the case of distorted geometry and different type of ligands, the trigonal bi- and pyramidal complexes do not exhibit zero-field FSG. Exceptions are the cases with strongly elongated and compressed bonds.

It is very essential to stress that even within such a degenerate ground state (see Figure 10b), the probability to observe unquenched orbital angular momentum equals zero. That results from the absence of virtual orbitals. Therefore, to some extent, the unquenching may be allowed in 3d systems with fewer than eight electrons and a low coordination number. That is the case of $3d^{2}$ complexes of the same coordination. We obtain a very large ZFS underpinned by the occurrence of unquenched orbital angular momentum; see Table 1. To gain an insight on the physical aspects related to the occurrence of huge ZFS and its relation to MA/SIA, with particular discussion on the cases of trigonal bipyramidal $3d^{2}$ and $3d^{8}$ complexes, the reader may further consult Ref. [62] and the references therein. Hence, all zero-field magnetic excitations and anisotropy that may be experimentally observed in Ni${}^{2+}$ trigonal bipyramidal complexes cannot be exclusively related to the spin–orbit interactions under the given CF symmetry. Examples demonstrating the difficulties encountered in studying ZFS in $3d^{8}$ trigonal bipyramidal complexes can be found in Refs. [35,36,37,38]. It appears that the only way to have zero-field FSG related to the magnetic properties of these complexes is to account for additional electron correlations, whose origin may be traced back to the existence of a phase difference between the wave functions of both unpaired electrons. In other words, we have to constrain the orbital motion of these electrons.

Since the ground state at B=0 is highly degenerate, the considered complex does not possess a magnetic moment. However, due to the lack of ZFS under the action of an external magnetic field, we observe a genuine Zeeman splitting, see Figure 10c. Accordingly, it requires no more than 2–3 T to fully polarize the system’s magnetic moment at 1 K obtaining M=1.99. The calculated magnetization per single complex is shown in Figure 5. The observed paramagnetic response is further clearly seen from the temperature dependence of low-field susceptibility per single complex depicted in Figure 3 and the field dependence presented in Figure 4. Note that with the lack of ZFS, both the octahedral and trigonal bipyramidal complexes respond equally to the action of the externally applied magnetic field.

### 3.4. Tetrahedral

**Figure 11:**
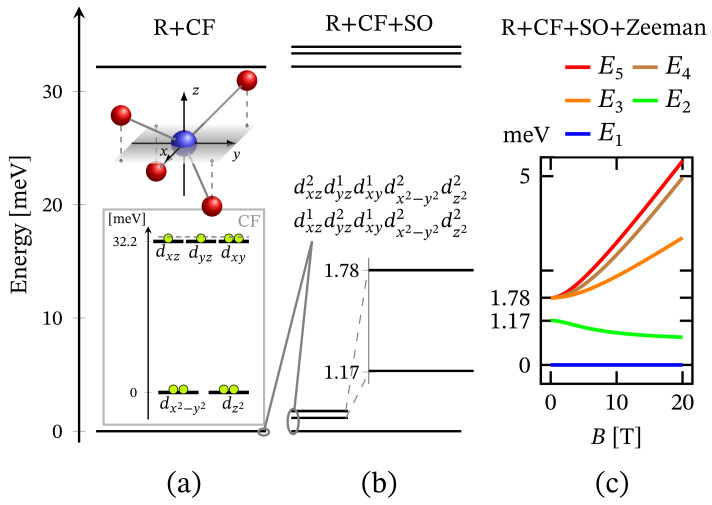
Low-lying energy level structure in the case of tetrahedral geometry. The values of the relevant parameters are given in Section 2.1. (**a**) Shows the splitting resulting from the effect of CF within the action of all repulsive Coulomb interactions. CF *d*-orbital splitting is illustrated in the inset, with electrons given as circles. The dashed line reminds that the considered configuration, with $d_{xy}$ core orbital, is one of the three possible. (**b**) Depicts the effect of spin–orbit interactions. (**c**) Presents the splitting of the low-lying energy levels resulting from the action of external magnetic field pointing along the *z* axis of the inner reference frame.

The zero-field energy spectrum obtained with Hamiltonian (1) adapted to the tetrahedral complex is shown in Figure 1c, with a low-lying energy level sequence depicted in Figure 11b. The ground and first excited states are associated to the configurations $d_{xz}^{1}d_{yz}^{2}d_{xy}^{1}d_{x^{2}-y^{2}}^{2}d_{z^{2}}^{2}$ and $d_{xz}^{2}d_{yz}^{1}d_{xy}^{1}d_{x^{2}-y^{2}}^{2}d_{z^{2}}^{2}$. The second excited energy level is 4-fold degenerate. Thus, for this specific type of geometry, we observe ZFS. Notice that in contrast to CF diagram depicted on the inset in Figure 11a the configuration $d_{xz}^{1}d_{yz}^{1}d_{xy}^{2}d_{x^{2}-y^{2}}^{2}d_{z^{2}}^{2}$ is no longer among the most energetically favorable ones and hence not related to the obtained FSG. It is positioned higher in the energy spectrum at around 580 meV since the repulsion between the electrons occupying $d_{xz}$ and $d_{yz}$ orbitals is much stronger than that occurring when one of the electrons occupy the $d_{xy}$ orbital.

**Figure 12:**
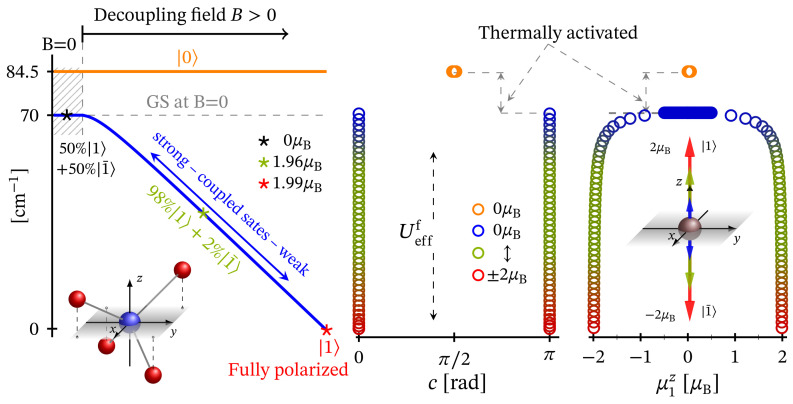
On the left panel, we show the evolution of the effective FSG as a function of the external magnetic field in the case of tetrahedral coordination along with the decoupling of the effective spin-one states |1〉 and $|\bar{1}\rangle$, coupled via spin–orbit interactions. The pathway to the state $|\bar{1}\rangle$ is a reflection taken with respect to the depicted energy axis. In the middle panel, we have the dependence of internal energy per single complex on the direction angle *c* taken between the principle axis and total magnetic moment expectation value. The calculation are performed at T=0.05 K. Further details are given in Table 1. The dependence of $U_{\mathrm{eff}}^{f}$ on the *z* component of the ground state magnetic moment is shown on the right panel.

The non-linear dependence of the corresponding FSG on the metal ion’s charge number is depicted in Figure 7. Notice that as a function of κ, FSG evolves linearly. We would like to stress that in spite of the contribution of spin–orbit interactions to the obtained zero-field fine structure, the presence of huge ZFS and giant MA is ruled out. Similar to the case of square planar complex, here, the magnitude of the anisotropy energy takes a non-zero value only under the influence of externally applied magnetic field. The corresponding magnetization-reversal barrier is approximately equal to 70 cm${}^{-1}$, see Figure 12 and Table 1. Thus, this complex does not exhibit a zero-field and non-thermally activated magnetization-reversal barrier. We would like to note that the magnetic behavior depicted in Figure 12 is qualitatively the same as that of the square planar complex, with the only difference being the energy barriers’ heights (see Table 1).

In the absence of an external magnetic field, the tetrahedral complex is non-magnetic. Similarly to the case of square planar geometry, the eigenstates are represented as a superposition of the spin-one quadrupole states (3) (consult also Equation (S7) in Supplementary materials). As a result, the applied magnetic field has a feeble influence on the complex magnetic properties and hence it resembles a diamagnet. In particular, for T≤10 K and $B=(0,0,B_{z})$, this complex is almost completely magnetized only when the external magnetic field is of magnitude greater than 30 T, see Figure 9. Furthermore, for $B\equiv B_{z}=30$ T and T=5 K, for the magnetization we get M=(0,0,1.9445). For the same temperature and magnetic field values, the gap between the ground and first excited energy levels is about 0.68 meV, with $\mu_{1}=\left(0,0,1.9352\right)$ and $\mu_{2}=\left(0,0,1.9919\right)$, respectively. In addition, for T>5 K, we observe the same behavior of the magnetization as displayed by the square planar complex. The saturation of the magnetization at T=15 K still holds. The weak response to the action of an externally applied magnetic field is further displayed in Figure 4 and Figure 8. We witness low susceptibility peaks corresponding to the negligible rate at which the magnetization changes with respect to the external magnetic field.

## 4. Summary

We performed a thorough theoretical investigation of FSG for a number of $3d^{8}$ coordination structures with Ni${}^{2+}$ metal centers. The study is based on the exact diagonalization technique, with active space limited to the number of all 3d orbitals [57]. The method considers all eight electrons as effectively localized around the metal ion and accounts for the contribution of all possible configurations within the used active space. We calculated the effect of CF, spin exchange and spin–orbit interactions on the low-lying energy level sequence for the octahedral, square planar, trigonal bipyramidal, tetrahedral, square pyramidal, trigonal pyramidal and trigonal planar geometries. Here, we present detailed results for the octahedral, trigonal bipyramidal, square planar, and tetrahedral complexes. Owing to the symmetry, results for the remaining structures may be easily obtained. To be complementary, the results for the zero-field FSG and low-temperature energy barriers of all studied complexes are compared to their counterparts in the case of V${}^{3+}$ systems.

We showed that without imposing constraints on the electrons’ degrees of freedom, the calculations show no signs of huge ZFS or giant MA in any of the Ni${}^{2+}$ complexes under consideration. This remains true even for a distorted geometry. Moreover, as a result of the highly occupied 3d subshell, the expectation values of the orbital magnetic moment in all the considered cases equal zero (see Table 1). Hence, no unquenched orbital angular momentum is observed. The results and their analysis clearly show the absence of zero-field FSG in the case of ideal geometries, where the ligands reside along the $d_{z^{2}}$ orbital. Two such complexes are the octahedral and the trigonal bipyramidal one. However, depending on the metal–ligand bond distances, a small to moderate ZFS is observed in the case of square and trigonal planar, tetrahedral and square pyramidal complexes. In contrast, with only two electrons in its 3d subshell, the Ni${}^{2+}$ spin-one equivalent V${}^{3+}$ may exhibit both large ZFS and unquenched orbital angular momentum. The results are summarized in Table 1.

None of the studied $3d^{8}$ systems exhibiting FSG demonstrates a zero-field anisotropy behavior. It appears that for these complexes, the dynamics between both spin-one fully polarized magnetic states is completely governed by a quantum tunneling of magnetization. In other words, the absence of the magnetization-reversal barrier at B=0 is a result of the uniform probability distribution of mutually opposite spin-one magnetic states in the superposition representing the ground state and low-lying excited states. Thus, in order to unravel the underlying anisotropy potential, we introduce an external magnetic field to the system and calculate the minimal energy separating both spin-one fully polarized magnetic states. The low-temperature field induced barriers’ heights are given in the last column on the right-hand side of Table 1. The calculated profile of the field induced magnetization-reversal barrier $U_{\mathrm{eff}}^{f}$ in the case of tetrahedral coordination is depicted in Figure 12. The barriers’ profiles of the square and trigonal planar and square pyramidal Ni${}^{2+}$ complexes are qualitatively identical. Quantitatively, their barriers vary only by height. For all aforementioned complexes, with barriers’ values listed in Table 1, the calculation shows that the quantum tunneling of magnetization could not be completely suppressed.

The effect of the externally applied magnetic field in FSG for the octahedral, trigonal bipyramidal, square planar and tetrahedral complexes is also reported. The latter two structures demonstrate remarkable resilience to the action of the external magnetic field as a result of the formation of very stable spin quadrupoles (3). In contrast, because of the absence of ZFS, the octahedral and trigonal bipyramidal complexes show isotropic behavior.

The investigation on all Ni${}^{2+}$ complexes unambiguously demonstrates that a non-trivial FSG does not arise from the action of CF and spin–orbit interactions alone. In other words, the spin–orbit coupling cannot lead to huge ZFS since on average, almost all of the corresponding matrix elements vanish (see Supplementary materials, Equation (S5)). Exploring all probable causes that may lead to the occurrence of huge ZFS in the considered complexes, we conclude that the only possible way is to confine the degrees of freedom of the electrons occupying active orbitals. In other words, we restrict their mutual orbital motion, which includes imposing a constraint over their phases. As a consequence, we observe a significant influence of the direct exchange interactions on the low-lying energy level spectrum. Hence, the results suggest that the spin exchange is the auxiliary mechanism to the spin–orbit coupling that leads to non-trivial ZFS. In particular, in conjunction with the action of CF and spin–orbit interactions the processes of direct exchange act in favor of the singlet configurations by mixing them with the triplet ones related to the ground state. As a result of the probability interference, the overall fine splitting increases significantly, reaching enormous values attaining up to hundreds of cm${}^{-1}$. That, furthermore, give rise to the coexistence of singlet-triplet and triplet-triplet magnetic excitations. The consideration of constraints and their impact over the FGS of $3d^{8}$ complexes merits a separate study that is beyond the scope of the present investigation and will be discussed elsewhere.

Besides the studied Ni${}^{2+}$-based complexes, the mathematical framework presented in Section 2.1 is applicable to arbitrary $3d^{8}$ systems. This includes molecular magnets and low-dimensional systems composed of well-isolated Cu${}^{3+}$, Co${}^{1-}$, Fe${}^{2-}$, etc., metal centers.

## Figures and Tables

**Table 1:** The zero-field FSG, magnitude of the spin $\mu_{1,s}$ and orbital $\mu_{1,l}$ magnetic moments at the ground state, zero-field $U_{\mathrm{eff}}$ and field induced $U_{\mathrm{eff}}^{f}$ magnetization-reversal barriers at T<0.1 K of some conventional V${}^{3+}$ and Ni${}^{2+}$ complexes. For all systems, the calculation are performed assuming bond distances of 2 Å, ligands’ charge numbers and orbital reduction factor equal to unity. The symbol “–” means not defined, i.e., it does not occur. The asterisk denotes that the corresponding *z* components take negative values. The double asterisk indicates that the barrier’s height can be increased to the given value due to fast unquenching.

	Overall ZFS [cm${}^{-1}$]		$\mu_{1,s}$ [$\mu_{B}$]		$\mu_{1,l}$ [$\mu_{B}$]		$U_{\mathrm{eff}}$ [cm${}^{-1}$]		$U_{\mathrm{eff}}^{f}$ [cm${}^{-1}$]	
**Complexes**	**V** ${}^{3+}$	**Ni** ${}^{2+}$	**V** ${}^{3+}$	**Ni** ${}^{2+}$	**V** ${}^{3+}$	**Ni** ${}^{2+}$	**V** ${}^{3+}$	**Ni** ${}^{2+}$	**V** ${}^{3+}$	**Ni** ${}^{2+}$
Octahedral	123	–	$0.153{\;}^{*}$	0	0.106	0	0.033	–	81	–
Square pyramidal	136	14.5	$0.138{\;}^{*}$	0	0.095	0	0.077	0	105	70
Square planar	12.9	15	$0.002{\;}^{*}$	0	0.003	0	0.016	0	0.2	72
Trigonal bipyramidal	278	–	$1.348{\;}^{*}$	0	2.031	0	0.074	–	169	–
Trigonal pyramidal	288	–	$1.773{\;}^{*}$	0	2.733	0	0.537	–	209	–
Trigonal planar	154	18.2	0	0	0	0	0	0	$165{\;}^{**}$	88
Tetrahedral	1.4	14.5	0	0	0	0	0	0	0.21	70

## Data Availability

The data generated within this research is included in the paper and Supplementary materials.

## Supplementary Materials

The following supporting information can be downloaded at: https://www.mdpi.com/article/10.3390/molecules27248887/s1.
