# Supplementary material for: Fine Structure and the Huge Zero-Field Splitting in Ni2+ Complexes
Source: Molecules. 2022 Dec 14;27(24):8887. doi: 10.3390/molecules27248887 (PMC9784865; doi:10.3390/molecules27248887)
Supplement: Supplementary file 1 [file molecules-27-08887-s001.zip › molecules-2086444-supplementary.pdf]

Supplementary Materials: Fine structure and the huge zero-field splitting in Ni^{2+} complexes

Miroslav Georgiev * and Hassan Chamati *

S1. Initial basis states

Since the active space associated to the Hamiltonian under consideration is restricted to the number of all five $3d$ orbitals and, moreover, two electrons are unpaired, we end up with forty five quantum basis states composed of ten triplets and fifteen singlets. Thus, using the bra-ket notation we have

$$
\begin{aligned}
|\phi_{1,s,m}\rangle &\equiv |\psi_{1,s,m}\rangle|\psi_{13,0,0}\rangle|\psi_{14,0,0}\rangle|\psi_{15,0,0}\rangle,\\
|\phi_{2,s,m}\rangle &\equiv |\psi_{2,s,m}\rangle|\psi_{12,0,0}\rangle|\psi_{14,0,0}\rangle|\psi_{15,0,0}\rangle,\\
|\phi_{3,s,m}\rangle &\equiv |\psi_{3,s,m}\rangle|\psi_{12,0,0}\rangle|\psi_{13,0,0}\rangle|\psi_{15,0,0}\rangle,\\
|\phi_{4,s,m}\rangle &\equiv |\psi_{4,s,m}\rangle|\psi_{12,0,0}\rangle|\psi_{13,0,0}\rangle|\psi_{14,0,0}\rangle,\\
|\phi_{5,s,m}\rangle &\equiv |\psi_{5,s,m}\rangle|\psi_{11,0,0}\rangle|\psi_{14,0,0}\rangle|\psi_{15,0,0}\rangle,\\
|\phi_{6,s,m}\rangle &\equiv |\psi_{6,s,m}\rangle|\psi_{11,0,0}\rangle|\psi_{13,0,0}\rangle|\psi_{15,0,0}\rangle,\\
|\phi_{7,s,m}\rangle &\equiv |\psi_{7,s,m}\rangle|\psi_{11,0,0}\rangle|\psi_{13,0,0}\rangle|\psi_{14,0,0}\rangle,\\
|\phi_{8,s,m}\rangle &\equiv |\psi_{8,s,m}\rangle|\psi_{11,0,0}\rangle|\psi_{12,0,0}\rangle|\psi_{15,0,0}\rangle,\\
|\phi_{9,s,m}\rangle &\equiv |\psi_{9,s,m}\rangle|\psi_{11,0,0}\rangle|\psi_{12,0,0}\rangle|\psi_{14,0,0}\rangle,\\
|\phi_{10,s,m}\rangle &\equiv |\psi_{10,s,m}\rangle|\psi_{11,0,0}\rangle|\psi_{12,0,0}\rangle|\psi_{13,0,0}\rangle,
\end{aligned}
\tag{S1a}
$$

and for the singlet states with only core orbitals, we have

$$
\begin{aligned}
|\phi_{11,0,0}\rangle &\equiv |\psi_{11,0,0}\rangle|\psi_{12,0,0}\rangle|\psi_{13,0,0}\rangle|\psi_{14,0,0}\rangle,\\
|\phi_{12,0,0}\rangle &\equiv |\psi_{12,0,0}\rangle|\psi_{11,0,0}\rangle|\psi_{13,0,0}\rangle|\psi_{15,0,0}\rangle,\\
|\phi_{13,0,0}\rangle &\equiv |\psi_{13,0,0}\rangle|\psi_{11,0,0}\rangle|\psi_{14,0,0}\rangle|\psi_{15,0,0}\rangle,\\
|\phi_{14,0,0}\rangle &\equiv |\psi_{14,0,0}\rangle|\psi_{11,0,0}\rangle|\psi_{12,0,0}\rangle|\psi_{15,0,0}\rangle,\\
|\phi_{15,0,0}\rangle &\equiv |\psi_{15,0,0}\rangle|\psi_{12,0,0}\rangle|\psi_{13,0,0}\rangle|\psi_{14,0,0}\rangle,
\end{aligned}
\tag{S1b}
$$

where $s = 0, 1$ and $m = \pm s$ are the total effective spin and magnetic quantum numbers, respectively.

In particular, for all $i = 1, \ldots, 15$, the states $|\psi_{i,s,m}\rangle$ in (S1) are given by

$$
\begin{aligned}
|\psi_{1,s,m}\rangle &\equiv \tfrac{1}{\sqrt{2}}\big(|d_{xz}, d_{yz}\rangle + (-1)^s|d_{yz}, d_{xz}\rangle\big)|s, m\rangle,\\
|\psi_{2,s,m}\rangle &\equiv \tfrac{1}{\sqrt{2}}\big(|d_{xz}, d_{xy}\rangle + (-1)^s|d_{xy}, d_{xz}\rangle\big)|s, m\rangle,\\
|\psi_{3,s,m}\rangle &\equiv \tfrac{1}{\sqrt{2}}\big(|d_{xz}, d_{x^2-y^2}\rangle + (-1)^s|d_{x^2-y^2}, d_{xz}\rangle\big)|s, m\rangle,\\
|\psi_{4,s,m}\rangle &\equiv \tfrac{1}{\sqrt{2}}\big(|d_{xz}, d_{z^2}\rangle + (-1)^s|d_{z^2}, d_{xz}\rangle\big)|s, m\rangle,\\
|\psi_{5,s,m}\rangle &\equiv \tfrac{1}{\sqrt{2}}\big(|d_{yz}, d_{xy}\rangle + (-1)^s|d_{xy}, d_{yz}\rangle\big)|s, m\rangle,\\
|\psi_{6,s,m}\rangle &\equiv \tfrac{1}{\sqrt{2}}\big(|d_{yz}, d_{x^2-y^2}\rangle + (-1)^s|d_{x^2-y^2}, d_{yz}\rangle\big)|s, m\rangle,\\
|\psi_{7,s,m}\rangle &\equiv \tfrac{1}{\sqrt{2}}\big(|d_{yz}, d_{z^2}\rangle + (-1)^s|d_{z^2}, d_{yz}\rangle\big)|s, m\rangle,\\
|\psi_{8,s,m}\rangle &\equiv \tfrac{1}{\sqrt{2}}\big(|d_{xy}, d_{x^2-y^2}\rangle + (-1)^s|d_{x^2-y^2}, d_{xy}\rangle\big)|s, m\rangle,\\
|\psi_{9,s,m}\rangle &\equiv \tfrac{1}{\sqrt{2}}\big(|d_{xy}, d_{z^2}\rangle + (-1)^s|d_{z^2}, d_{xy}\rangle\big)|s, m\rangle,\\
|\psi_{10,s,m}\rangle &\equiv \tfrac{1}{\sqrt{2}}\big(|d_{x^2-y^2}, d_{z^2}\rangle + (-1)^s|d_{z^2}, d_{x^2-y^2}\rangle\big)|s, m\rangle,
\end{aligned}
\tag{S2a}
$$

and

$$\begin{aligned} |\psi_{11,0,0}\rangle &\equiv |d_{xz}, d_{xz}\rangle|0,0\rangle, \\ |\psi_{12,0,0}\rangle &\equiv |d_{yz}, d_{yz}\rangle|0,0\rangle, \\ |\psi_{13,0,0}\rangle &\equiv |d_{xy}, d_{xy}\rangle|0,0\rangle, \\ |\psi_{14,0,0}\rangle &\equiv |d_{x^2-y^2}, d_{x^2-y^2}\rangle|0,0\rangle, \\ |\psi_{15,0,0}\rangle &\equiv |d_{z^2}, d_{z^2}\rangle|0,0\rangle. \end{aligned} \tag{S2b}$$

In general, each state in (S1) can be written as a superposition of some particular Slater determinants. For example, assume we have four electrons and three orbitals, with single electron wave function $\varphi_{n,m_i}(\mathbf{r}_i)$, where m_i is the corresponding spin magnetic quantum number, $i = 1, \ldots, 4$ and $n = 1, 2, 3$. Then, the total spin triplet wave function, describing a case in which the 2-nd and 3-rd orbitals are active and the 1-st one is a core orbital, reads

$$\begin{aligned} \Psi_{s,m}(\mathbf{r}_1, \ldots, \mathbf{r}_4) \equiv \frac{1}{\sqrt{6}}\Big[&\Psi^{2,3}_{s,m}(\mathbf{r}_1,\mathbf{r}_2)\Phi^1_{0,0}(\mathbf{r}_3,\mathbf{r}_4) - \Psi^{2,3}_{s,m}(\mathbf{r}_1,\mathbf{r}_3)\Phi^1_{0,0}(\mathbf{r}_2,\mathbf{r}_4) + \Psi^{2,3}_{s,m}(\mathbf{r}_1,\mathbf{r}_4)\Phi^1_{0,0}(\mathbf{r}_2,\mathbf{r}_3) \\ &+ \Psi^{2,3}_{s,m}(\mathbf{r}_2,\mathbf{r}_3)\Phi^1_{0,0}(\mathbf{r}_1,\mathbf{r}_4) - \Psi^{2,3}_{s,m}(\mathbf{r}_2,\mathbf{r}_4)\Phi^1_{0,0}(\mathbf{r}_1,\mathbf{r}_3) + \Psi^{2,3}_{s,m}(\mathbf{r}_3,\mathbf{r}_4)\Phi^1_{0,0}(\mathbf{r}_1,\mathbf{r}_2)\Big], \end{aligned}$$

where using the "up" and "down" notation, with $m_i \in \{\uparrow, \downarrow\}\ \forall i$, we have

$$\Phi^1_{0,0}(\mathbf{r}_i,\mathbf{r}_j) \equiv \frac{1}{\sqrt{2}}\left[\varphi_{1,\uparrow}(\mathbf{r}_i)\varphi_{1,\downarrow}(\mathbf{r}_j) - \varphi_{1,\downarrow}(\mathbf{r}_i)\varphi_{1,\uparrow}(\mathbf{r}_j)\right]$$

and for example

$$\Psi^{2,3}_{1,0}(\mathbf{r}_i,\mathbf{r}_j) \equiv \frac{1}{\sqrt{4}}\Big[\varphi_{2,\uparrow}(\mathbf{r}_i)\varphi_{3,\downarrow}(\mathbf{r}_j) + \varphi_{2,\downarrow}(\mathbf{r}_i)\varphi_{3,\uparrow}(\mathbf{r}_j) - \varphi_{3,\uparrow}(\mathbf{r}_i)\varphi_{2,\downarrow}(\mathbf{r}_j) - \varphi_{3,\downarrow}(\mathbf{r}_i)\varphi_{2,\uparrow}(\mathbf{r}_j)\Big].$$

Hence, according to the applied bra-ket denotations in (S1), we have $\Psi_{s,m}(\mathbf{r}_1, \ldots, \mathbf{r}_4) \rightarrow |\Psi_{s,m}\rangle|\Phi_{0,0}\rangle$.

S2. Coulomb terms

The matrix elements corresponding to the Coulomb interactions accounted for by the operator $\hat{U}_\mathrm{R}$ (see Equation (2a)) are calculated using series expansions up to the 4-th order. Starting with the basis states (S1) for all m, we set $\langle\phi_{i,s,m}|\hat{U}_\mathrm{R}|\phi_{i,s,m}\rangle = \mathcal{E}_{i,s}$ to obtain

$$\begin{aligned} \mathcal{E}_{1,s} &= F_1 - \delta_{1s}F_{10} + X_1, & \mathcal{E}_{2,s} &= F_4 + (-1)^s F_7 + X_2, \\ \mathcal{E}_{3,s} &= F_4 + (-1)^s F_7 + X_3, & \mathcal{E}_{4,s} &= F_5 + (-1)^s F_8 + X_4, \\ \mathcal{E}_{5,s} &= F_4 + (-1)^s F_7 + X_5, & \mathcal{E}_{6,s} &= F_4 + (-1)^s F_7 + X_6, \\ \mathcal{E}_{7,s} &= F_5 + (-1)^s F_8 + X_7, & \mathcal{E}_{8,s} &= F_2 - \delta_{1s}F_{11} + X_8, \\ \mathcal{E}_{9,s} &= F_6 + (-1)^s F_9 + X_9, & \mathcal{E}_{10,s} &= F_6 + (-1)^s F_9 + X_{10}, \\ \mathcal{E}_{11,0} &= F_1 + \tfrac{1}{2}F_{10} + X_{11}, & \mathcal{E}_{12,0} &= F_1 + \tfrac{1}{2}F_{10} + X_{12}, \\ \mathcal{E}_{13,0} &= F_2 + \tfrac{1}{2}F_{11} + X_{13}, & \mathcal{E}_{14,0} &= F_2 + \tfrac{1}{2}F_{11} + X_{14}, \\ \mathcal{E}_{15,0} &= F_3 + X_{15}. \end{aligned} \tag{S3}$$

Let us point out that for all i, the functions $F_i \equiv F_i(Z)$ and $X_i \equiv X_i(Z)$ depend implicitly on the phase difference between the orbital states of the corresponding electrons. Therefore, some local phases should be taken into account during the calculation of the direct exchange integrals. That is required in order to ensure the correspondence between the results obtained from the application of free-ion and CF basis states. Otherwise, we will loose consistency and for example F_7 could be calculated as $\frac{1}{2}F_7 + \frac{1}{4}F_{11}$.

The energy values given in (S3) are represented only as linear functions of the metal ion's charge number. For all i, the explicit representation of functions $F_i(Z)$ and $X_i(Z)$ are given by

$$F_i = a_i \frac{\gamma Z}{r_{\mathrm{B}}}, \tag{S4}$$

where r_{B} is the Bohr radius and

$$\begin{aligned}
a_1 &= \frac{42601}{483840}, & a_2 &= \frac{43459}{483840}, & a_3 &= \frac{29731}{322560}, & a_4 &= \frac{81211}{967680}, \\
a_5 &= \frac{27833}{322560}, & a_6 &= \frac{26689}{322560}, & a_7 &= \frac{5707}{967680}, & a_8 &= \frac{949}{322560}, \\
a_9 &= \frac{169}{35840}, & a_{10} &= \frac{3991}{483840}, & a_{11} &= \frac{65}{13824}.
\end{aligned}$$

In terms of the functions F_i in (S4), for $X_i = X_i(Z)$, we get

$$\begin{aligned}
X_1 &= 6F_2 - F_{11} + F_3 + 8F_4 + 4F_5 + 8F_6, \\
X_2 &= 3F_1 - \frac{1}{2}F_{10} + 3F_2 - \frac{1}{2}F_{11} + F_3 + 8F_4 + 6F_5 + 6F_6, \\
X_4 &= 3F_1 - \frac{1}{2}F_{10} + 6F_2 - F_{11} + 12F_4 + 2F_5 + 4F_6, \\
X_8 &= 6F_1 - F_{10} + F_3 + 8F_4 + 8F_5 + 4F_6, \\
X_9 &= 6F_1 - F_{10} + 3F_2 - \frac{1}{2}F_{11} + 12F_4 + 4F_5 + 2F_6, \\
X_{11} &= 5F_1 - \frac{3}{2}F_{10} + 6F_2 - F_{11} + 16F_4, \\
X_{12} &= 5F_1 - \frac{3}{2}F_{10} + F_2 + \frac{1}{2}F_{11} + F_3 + 8F_4 + 8F_5 + 4F_6, \\
X_{13} &= F_1 + \frac{1}{2}F_{10} + 5F_2 - \frac{3}{2}F_{11} + F_3 + 8F_4 + 4F_5 + 8F_6, \\
X_{14} &= 6F_1 - F_{10} + F_3 + 8F_4 + 8F_5 + 4F_6, \\
X_{15} &= F_1 + \frac{1}{2}F_{10} + 6F_2 - F_{11} + 8F_4 + 4F_5 + 8F_6,
\end{aligned}$$

where $X_6 = X_5 = X_3 = X_2$, $X_7 = X_4$ and $X_{10} = X_9$.

S3. CF terms

Since the $3d$ subshell is filled to a large extent, from Equation (2b) we obtain only forty off-diagonal matrix elements. They are related to the pure singlet states, or $\langle \phi_{i,s,m} | \hat{U}_{\mathrm{CF}} | \phi_{j,s',m'} \rangle = \delta_{0s}\delta_{s's}U_{i,s,m;j,s',m'}$. Hence, denoting the diagonal entities by $U_{i,s,m}$, for all m, we have

$$\begin{aligned}
U_{1,s,m} &= U_{xz} + U_{yz} + 2U_{xy} + 2U_{x^2-y^2} + 2U_{z^2}, \\
U_{2,s,m} &= U_{xz} + U_{xy} + 2U_{yz} + 2U_{x^2-y^2} + 2U_{z^2}, \\
U_{3,s,m} &= U_{xz} + U_{x^2-y^2} + 2U_{yz} + 2U_{xy} + 2U_{z^2}, \\
U_{4,s,m} &= U_{xz} + U_{z^2} + 2U_{yz} + 2U_{xy} + 2U_{x^2-y^2}, \\
U_{5,s,m} &= U_{yz} + U_{xy} + 2U_{xz} + 2U_{x^2-y^2} + 2U_{z^2}, \\
U_{6,s,m} &= U_{yz} + U_{x^2-y^2} + 2U_{xz} + 2U_{xy} + 2U_{z^2}, \\
U_{7,s,m} &= U_{yz} + U_{z^2} + 2U_{xz} + 2U_{xy} + 2U_{x^2-y^2}, \\
U_{8,s,m} &= U_{xy} + U_{x^2-y^2} + 2U_{xz} + 2U_{yz} + 2U_{z^2}, \\
U_{9,s,m} &= U_{xy} + U_{z^2} + 2U_{xz} + 2U_{yz} + 2U_{x^2-y^2}, \\
U_{10,s,m} &= U_{x^2-y^2} + U_{z^2} + 2U_{xz} + 2U_{yz} + 2U_{xy}
\end{aligned}$$

and

$$
\begin{aligned}
U_{11,0,0} &= 2U_{xz} + 2U_{yz} + 2U_{xy} + 2U_{x^2-y^2},\\
U_{12,0,0} &= 2U_{xz} + 2U_{yz} + 2U_{xy} + 2U_{z^2},\\
U_{13,0,0} &= 2U_{xz} + 2U_{xy} + 2U_{x^2-y^2} + 2U_{z^2},\\
U_{14,0,0} &= 2U_{xz} + 2U_{yz} + 2U_{x^2-y^2} + 2U_{z^2},\\
U_{15,0,0} &= 2U_{yz} + 2U_{xy} + 2U_{x^2-y^2} + 2U_{z^2}.
\end{aligned}
$$

All CF diagrams shown in the inset of Figures 2 (a), 6 (a), 10 (a) and 11 (a), depict the configuration related to the term $U_{i,s,m}$ having the lowest energy value. For the off-diagonal ones we apply the shorthand designation $U_{i,j,0,0}$, obtaining

$$
\begin{aligned}
U_{1,13,0,0} &= \sqrt{2}U_{xz,yz}, & U_{1,15,0,0} &= \sqrt{2}U_{xz,yz},\\
U_{2,14,0,0} &= \sqrt{2}U_{xz,xy}, & U_{2,15,0,0} &= \sqrt{2}U_{xz,xy},\\
U_{3,12,0,0} &= \sqrt{2}U_{xz,x^2-y^2}, & U_{3,15,0,0} &= \sqrt{2}U_{xz,x^2-y^2},\\
U_{4,11,0,0} &= \sqrt{2}U_{xz,z^2}, & U_{4,15,0,0} &= \sqrt{2}U_{xz,z^2},\\
U_{5,13,0,0} &= \sqrt{2}U_{yz,xy}, & U_{5,14,0,0} &= \sqrt{2}U_{yz,xy},\\
U_{6,12,0,0} &= \sqrt{2}U_{yz,x^2-y^2}, & U_{6,13,0,0} &= \sqrt{2}U_{yz,x^2-y^2},\\
U_{7,11,0,0} &= \sqrt{2}U_{yz,z^2}, & U_{7,13,0,0} &= \sqrt{2}U_{yz,z^2},\\
U_{8,12,0,0} &= \sqrt{2}U_{xy,x^2-y^2}, & U_{8,14,0,0} &= \sqrt{2}U_{xy,x^2-y^2},\\
U_{9,11,0,0} &= \sqrt{2}U_{xy,z^2}, & U_{9,14,0,0} &= \sqrt{2}U_{xy,z^2},\\
U_{10,11,0,0} &= \sqrt{2}U_{x^2-y^2,z^2}, & U_{10,12,0,0} &= \sqrt{2}U_{x^2-y^2,z^2}.
\end{aligned}
$$

S4. Spin-orbit terms

Due to the almost completely filled subshell, nearly all average values of the spin-orbit interactions (see Equation (2c)) vanish. In total, we have fifty six off-diagonal elements, all exclusively related to the singlet states (S1b). For $\langle \phi_{i,s,m}|\hat{U}_{\mathrm{SO}}|\phi_{j,s',m'}\rangle = Y_{i,s,m;j,s',m'}$, we have

$$
\begin{aligned}
Y_{1,1,0;13,0,0} &= -i\sqrt{2}\eta, & Y_{1,1,0;15,0,0} &= -i\sqrt{2}\eta,\\
Y_{8,1,0;12,0,0} &= i2\sqrt{2}\eta, & Y_{8,1,0;14,0,0} &= i2\sqrt{2}\eta,\\
Y_{4,1,\pm1;11,0,0} &= -\sqrt{3}\eta, & Y_{7,1,\pm1;11,0,0} &= \pm i\sqrt{3}\eta,\\
Y_{3,1,\pm1;12,0,0} &= \eta, & Y_{6,1,\pm1;12,0,0} &= \pm i\eta,\\
Y_{5,1,\pm1;13,0,0} &= \eta, & Y_{6,1,\pm1;13,0,0} &= \pm i\eta,\\
Y_{7,1,\pm1;13,0,0} &= \pm i\sqrt{3}\eta, & Y_{2,1,\pm1;14,0,0} &= \mp i\eta,\\
Y_{5,1,\pm1;14,0,0} &= \eta, & Y_{2,1,\pm1;15,0,0} &= \mp i\eta,\\
Y_{3,1,\pm1;15,0,0} &= i\eta, & Y_{4,1,\pm1;15,0,0} &= -\sqrt{3}\eta,
\end{aligned}
\tag{S5}
$$

where $\eta = \kappa\zeta/2$, with κ and ζ being the covalence coefficient and spin-orbit coupling. Therefore, we would like to stress that if one does not account for the complete set of singlet states, in particular (S1b), the spin-orbit interactions will have no contribution to the resulting energy spectrum and FSG will not be observed. That is also true if we perform the calculations using the free-ion basis states.

S5. Zeeman terms

Within the basis states (S1) the diagonal matrix elements associated to the Zeeman term $\hat{U}_{\mathrm{Z}}$ (see Equation (2d)) depend only on the spin degrees of freedom, whence $Z_{i,s,m} = -g_e\mu_{\mathrm{B}}mB_z$. We further have forty off-diagonal entities corresponding to the spin component of the wave function that are a mixture between $m = 0$ and $m = \pm1$ states. Thus, the non-conjugate ones are written as

$$
Z_{i,1,\pm1;i,1,0} = -g_e\mu_{\mathrm{B}}(B_x \mp iB_y)/\sqrt{2},
$$

where $i = 1, \ldots, 10$. Due to the highly filled subshell, all remaining matrix elements result from the mixing of only spin singlet states. Taking into account the substitution $Z_{i,s,m;j,s,m} \to Z_{i,j,s,m}$, we get

$$\begin{aligned}
Z_{13,1,0,0} &= i\sqrt{2}\mu_B B_z, & Z_{15,1,0,0} &= -i\sqrt{2}\mu_B B_z,\\
Z_{14,2,0,0} &= -i\sqrt{2}\mu_B B_x, & Z_{15,2,0,0} &= i\sqrt{2}\mu_B B_x,\\
Z_{12,3,0,0} &= i\sqrt{2}\mu_B B_y, & Z_{15,3,0,0} &= -i\sqrt{2}\mu_B B_y,\\
Z_{11,4,0,0} &= -i\sqrt{6}\mu_B B_y, & Z_{15,4,0,0} &= i\sqrt{6}\mu_B B_y,\\
Z_{14,5,0,0} &= i\sqrt{2}\mu_B B_y, & Z_{13,5,0,0} &= -i\sqrt{2}\mu_B B_y,\\
Z_{12,6,0,0} &= i\sqrt{2}\mu_B B_x, & Z_{13,6,0,0} &= -i\sqrt{2}\mu_B B_x,\\
Z_{11,7,0,0} &= i\sqrt{6}\mu_B B_x, & Z_{13,7,0,0} &= -i\sqrt{6}\mu_B B_x,\\
Z_{12,8,0,0} &= -i2\sqrt{2}\mu_B B_z, & Z_{14,8,0,0} &= i2\sqrt{2}\mu_B B_z.
\end{aligned}$$

S6. Energy spectra

The values of all roots from the characteristic polynomial of the total effective matrix obtained from Equation (1) for the main coordination geometries, with matrix elements presented in Sections S2.–S5., are given in eV hereafter.

Consider the total zero-field energy spectrum of the octahedral complex depicted in Figure 1 (a). Starting from the highest energy level on the left moving down to the ground state energy level on the bottom right, we have

$$\{4.77057, 4.76879, 4.76796, 4.62521, 4.625, 3.9963, 3.9963, 3.92394, 3.92394, 3.92394, 3.73092, 3.73092, 3.41725, 3.15187, 3.0795, 2.38857, 2.38857, 2.37905, 1.80952, 1.80952, 1.80952, 1.80952, 1.80673, 1.80088, 0.723781, 0.723781, 0.722419, 0.144729, 0.144729, 0.144729, 0.144729, 0.144138, 0.142944, 0.0723645, 0.0723645, 0.0723645, 0.0723645, 0.0723645, 0.0723645, 0.0723645, 0.071772, 0.0705609, 0, 0, 0\}.$$

The first three roots, bottom right, are of identical values due to the 3-fold degeneracy of the ground state electron configuration, see Section S71.

Similarly, for the square planar and trigonal bipyramidal complexes with energy spectra depicted accordingly in Figure 1 (b) and (c), we get

$$\{5.24645, 5.05616, 5.05489, 4.34219, 4.19747, 4.18513, 4.18513, 3.9258, 3.9258, 3.85344, 3.85344, 3.7041, 3.49608, 3.24873, 3.17636, 2.26373, 2.26373, 2.26373, 2.26373, 2.26078, 2.25526, 1.96072, 1.96072, 1.9512, 1.01063, 1.01063, 1.00927, 0.169226, 0.169226, 0.169226, 0.096861, 0.096861, 0.096861, 0.0742292, 0.0742292, 0.0742292, 0.0742292, 0.07368, 0.0723869, 0.00186467, 0.00186467, 0.00186467, 0.00186467, 0.00131479, 0\}$$

and

$$\{4.78827, 4.77875, 4.65857, 4.65714, 4.46676, 4.00989, 3.94769, 3.94769, 3.94769, 3.94769, 3.59635, 3.59635, 3.30643, 3.0795, 3.0795, 2.47452, 2.47452, 2.46501, 1.67495, 1.67495, 1.67495, 1.67495, 1.67219, 1.66629, 0.612964, 0.612964, 0.611603, 0.0961136, 0.0961136, 0.0961136, 0.0961136, 0.0961136, 0.0961136, 0.0961136, 0.0961136, 0.0960227, 0.0953776, 0.0944646, 0.0938163, 0, 0, 0, 0, 0, 0\},$$

respectively. For the tetrahedral one, shown in Figure 1 (d), we have

$$\{4.69162, 4.69128, 4.62732, 4.62564, 4.62514, 3.88553, 3.88553, 3.88553, 3.85337, 3.85337, 3.69252, 3.69252, 3.27432, 3.14563, 3.11347, 2.35017, 2.35017, 2.34066, 1.77112, 1.77112, 1.77112, 1.77112, 1.76823, 1.76259, 0.580852, 0.580852, 0.579492, 0.0661247, 0.0661247, 0.0661247, 0.0339627, 0.0339627, 0.0339627, 0.0339627, 0.0339627, 0.0339627, 0.0339627, 0.0333725, 0.032189, 0.00180066, 0.00180066, 0.00180066, 0.00180066, 0.00119307, 0\}.$$

S7. Energy eigenstates related to FSG

For the sake of clarity, the probability coefficients to all eigenstates are taken by rounding within 10^{-2}.

S71. Octahedral coordination

The ground state energy level is three fold degenerate. The corresponding eigenstates are

$$\begin{aligned}
|\Psi_1\rangle &= (0.08 - 0.12i)|\phi_{10,1,\bar{1}}\rangle + (0.06 - 0.46i)|\phi_{10,1,0}\rangle - (0.21 - 0.85i)|\phi_{10,1,1}\rangle,\\
|\Psi_2\rangle &= -(0.68 + 0.02i)|\phi_{10,1,\bar{1}}\rangle - (0.59 + 0.05i)|\phi_{10,1,0}\rangle - (0.42 + 0.03i)|\phi_{10,1,1}\rangle,\\
|\Psi_3\rangle &= -0.72|\phi_{10,1,\bar{1}}\rangle + 0.65|\phi_{10,1,0}\rangle + (0.23 + 0.08i)|\phi_{10,1,1}\rangle.
\end{aligned}$$

S72. Square planar coordination

For the first two energy eigenstates we obtain

$$\begin{aligned}
|\Psi_k\rangle =& (-1)^{k+1}0.49i|\phi_{3,1,\bar{1}}\rangle + (-1)^{k+1}0.49i|\phi_{3,1,1}\rangle + 0.49|\phi_{6,1,\bar{1}}\rangle - 0.49|\phi_{6,1,1}\rangle\\
&+ \sum_{n\geq 2} O(10^{-n})|\phi_{\dots}\rangle,
\end{aligned}$$

where $k = 1, 2$. The third state is given by the following superposition

$$\begin{aligned}
|\Psi_3\rangle =& -(0.08 - 0.01i)|\phi_{3,1,1}\rangle + (0.08 - 0.01i)|\phi_{3,1,\bar{1}}\rangle - (0.19 + 0.08i)|\phi_{6,1,1}\rangle\\
&- (0.19 + 0.08i)|\phi_{6,1,\bar{1}}\rangle + (0.87 - 0.39i)|\phi_{6,1,0}\rangle + O(10^{-3})|\phi_{\dots}\rangle.
\end{aligned}$$

The superposition of the 4-th and 5-th eigenstates is a combination of the same basis vectors. Thus,

$$\begin{aligned}
|\Psi_4\rangle =& -(0.15 + 0.06i)|\phi_{3,1,1}\rangle + (0.15 + 0.06i)|\phi_{3,1,\bar{1}}\rangle - (0.61 + 0.23i)|\phi_{6,1,1}\rangle\\
&- (0.61 + 0.23i)|\phi_{6,1,\bar{1}}\rangle - (0.28 - 0.13i)|\phi_{6,1,0}\rangle\\
|\Psi_5\rangle =& -(0.50 - 0.47i)|\phi_{3,1,1}\rangle + (0.50 - 0.47i)|\phi_{3,1,\bar{1}}\rangle + (0.13 - 0.11i)|\phi_{6,1,1}\rangle\\
&+ (0.13 - 0.11i)|\phi_{6,1,\bar{1}}\rangle - (0.05 - 0.02i)|\phi_{6,1,0}\rangle,\\
|\Psi_6\rangle =& |\phi_{3,1,0}\rangle.
\end{aligned}$$

S8. Trigonal bipyramidal coordination

In this case we have the highest degeneracy, we get

$$\begin{aligned}
|\Psi_1\rangle =& 0.06|\phi_{10,1,0}\rangle - (0.16 - 0.10i)|\phi_{10,1,\bar{1}}\rangle - (0.12 + 0.06i)|\phi_{10,1,1}\rangle - (0.30 + 0.79i)|\phi_{9,1,0}\rangle\\
&- (0.04 - 0.21i)|\phi_{9,1,\bar{1}}\rangle + (0.11 - 0.41i)|\phi_{9,1,1}\rangle,\\
|\Psi_2\rangle =& (-0.22 + 0.04i)|\phi_{10,1,0}\rangle + (0.126 + 0.027i)|\phi_{10,1,\bar{1}}\rangle + (0.53 + 0.09i)|\phi_{10,1,1}\rangle\\
&- (0.15 + 0.41i)|\phi_{9,1,0}\rangle + (0.15 + 0.04i)|\phi_{9,1,\bar{1}}\rangle - (0.13 - 0.64i)|\phi_{9,1,1}\rangle\\
|\Psi_3\rangle =& (-0.01 + 0.12i)|\phi_{10,1,0}\rangle - (0.63 - 0.15i)|\phi_{10,1,\bar{1}}\rangle - (0.32 - 0.39i)|\phi_{10,1,1}\rangle\\
&- (0.18 - 0.04i)|\phi_{9,1,0}\rangle - (0.27 + 0.08i)|\phi_{9,1,\bar{1}}\rangle + (0.17 + 0.41i)|\phi_{9,1,1}\rangle.
\end{aligned}$$

Furthermore,

$$
\begin{aligned}
|\Psi_4\rangle = &(-0.69+0.01i)|\phi_{10,1,0}\rangle - (0.10-0.10i)|\phi_{10,1,\bar{1}}\rangle + (0.21-0.22i)|\phi_{10,1,1}\rangle \\
&- (0.05-0.09i)|\phi_{9,1,0}\rangle - (0.57+0.05i)|\phi_{9,1,\bar{1}}\rangle - (0.14+0.21i)|\phi_{9,1,1}\rangle, \\
|\Psi_5\rangle = &(-0.65+0.06i)|\phi_{10,1,0}\rangle + (0.25-0.07i)|\phi_{10,1,\bar{1}}\rangle - (0.36-0.31i)|\phi_{10,1,1}\rangle \\
&+ (0.03-0.02i)|\phi_{9,1,0}\rangle + (0.40+0.06i)|\phi_{9,1,\bar{1}}\rangle + (0.33-0.05i)|\phi_{9,1,1}\rangle \\
|\Psi_6\rangle = &(0.1+0.13i)|\phi_{10,1,0}\rangle + (0.53+0.40i)|\phi_{10,1,\bar{1}}\rangle - (0.34+0.02i)|\phi_{10,1,1}\rangle \\
&- 0.19i|\phi_{9,1,0}\rangle - (0.28+0.53i)|\phi_{9,1,\bar{1}}\rangle - (0.05-0.14i)|\phi_{9,1,1}\rangle.
\end{aligned}
$$

S9. Tetrahedral coordination

The ground and first excited states are

$$
|\Psi_k\rangle = (-1)^k 0.49i|\phi_{2,1,\bar{1}}\rangle - (-1)^k 0.49i|\phi_{2,1,1}\rangle - 0.49|\phi_{5,1,\bar{1}}\rangle - 0.49|\phi_{5,1,1}\rangle + \sum_{n\geq 2} O\left(10^{-n}\right)|\phi_{\ldots}\rangle, \tag{S6}
$$

where $k = 1,2$. The remaining eigenstates related to the excited levels from the corresponding FSG are given by

$$
\begin{aligned}
|\Psi_3\rangle = &(0.02-0.67i)|\phi_{2,1,1}\rangle + (0.02-0.67i)|\phi_{2,1,\bar{1}}\rangle - 0.06|\phi_{5,1,0}\rangle + (0.22+0.03i)|\phi_{5,1,1}\rangle \\
&- (0.22+0.03i)|\phi_{5,1,\bar{1}}\rangle, \\
|\Psi_4\rangle = &(0.03+0.22i)|\phi_{2,1,1}\rangle + (0.03+0.22i)|\phi_{2,1,\bar{1}}\rangle - 0.06|\phi_{5,1,0}\rangle + 0.67|\phi_{5,1,1}\rangle - 0.67|\phi_{5,1,\bar{1}}\rangle, \\
|\Psi_5\rangle = &-0.02i|\phi_{2,1,1}\rangle - 0.02i|\phi_{2,1,\bar{1}}\rangle + (0.99-0.03i)|\phi_{5,1,0}\rangle + 0.05|\phi_{5,1,1}\rangle - 0.05|\phi_{5,1,\bar{1}}\rangle \\
|\Psi_6\rangle = &|\phi_{2,1,0}\rangle.
\end{aligned}
$$
